# OrthoClust: an orthology-based network framework for clustering data across multiple species

**DOI:** 10.1186/gb-2014-15-8-r100

**Published:** 2014-08-28

**Authors:** Koon-Kiu Yan, Daifeng Wang, Joel Rozowsky, Henry Zheng, Chao Cheng, Mark Gerstein

**Affiliations:** Program in Computational Biology and Bioinformatics, Yale University, New Haven, CT 06520 USA; Department of Molecular Biophysics and Biochemistry, Yale University, New Haven, CT 06520 USA; Department of Computer Science, Yale University, New Haven, CT 06520 USA; Department of Genetics, Dartmouth School of Medicine, Hanover, NH 03755 USA

## Abstract

Increasingly, high-dimensional genomics data are becoming available for many organisms.Here, we develop OrthoClust for simultaneously clustering data across multiple species. OrthoClust is a computational framework that integrates the co-association networks of individual species by utilizing the orthology relationships of genes between species. It outputs optimized modules that are fundamentally cross-species, which can either be conserved or species-specific. We demonstrate the application of OrthoClust using the RNA-Seq expression profiles of *Caenorhabditis elegans* and *Drosophila melanogaster* from the modENCODE consortium. A potential application of cross-species modules is to infer putative analogous functions of uncharacterized elements like non-coding RNAs based on guilt-by-association.

**Electronic supplementary material:**

The online version of this article (doi:10.1186/gb-2014-15-8-r100) contains supplementary material, which is available to authorized users.

## Background

Over the past decade, we have witnessed the burgeoning of comparative genomics. With the advancement of sequencing and other high-throughput techniques, ‘omics’-scale data have been generated in many species [1, 2]. Apart from genomic sequences, one can now compare two or more species in terms of their epigenome, regulome, transcriptome, interactome, and so on. As a result, computational frameworks that integrate such system-level data from different species are of particular interest. While different kinds of ‘omics’ scale data reflect different facets of a biological system, many of these high-dimensional data can be projected onto a network. For instance, the expression profiles of genes or the histone modification patterns in their upstream regions can be used to connect genes to form various co-association networks. Data from different species thus form species-specific networks that can, in principle, be integrated by incorporating evolutionary relationships.

For a set of genes, features associated with the topological properties of networks open additional windows to interpret their genomics features and annotation, among which the concept of network modules is particularly important from a systems biology perspective. Through identifying modules, one can reduce the complexity of a biological system by collapsing the large number of interconnections amongst its constituents into a smaller number of interactions between the modules [3, 4]. While different ‘omic’ data result in different networks, genes clustered together to form modules are likely to have a common biological role; for instance, being regulated by a common transcription factor, being part of a protein complex, or being presented in the same pathway. One of the most widely studied types of ‘omic’-scale data is genome-wide expression data. To analyze genome-wide expression profiles, network-based algorithms [5] together with approaches like hierarchical clustering [6], self-organized maps [7], spectral techniques [8] and superparamagnetic clustering [9] have been developed and extensively used since the dawn of the microarray era. While these methods have provided valuable biological insights, they were aimed at clustering within individual species only. To utilize the evolutionary information between species, a natural generalization that performs clustering across multiple species will be instructive, especially because the recent advancements in transcriptome profiling techniques like RNA-Seq have generated tremendous amounts of genome-wide expression data across many different species [10, 11].

Here we present OrthoClust, a novel network-based framework for clustering data across multiple species. OrthoClust integrates the networks of individual species using orthology relationships of genes between species. As connected genes within a species and orthologous pairs across species connect genes with the same function within and across species, respectively, OrthoClust naturally extends the idea of functional modules into a cross-species dimension. The essence of OrthoClust is a cost function for the detection of modules across species. We present a solution of the optimization problem by using simulated annealing. As expression data comprise one of the most important classes of 'omic-' data, we demonstrate OrthoClust using the genome-wide expression data of worm and fly generated by the modENCODE consortium, arriving at co-expression modules that range from being highly conserved to species-specific. We then compare the results with traditional single-species clustering and demonstrate the advantage of our approach. We further compare the conserved modules with results obtained from IsoRank [12], one of the state-of-the-art methods for network alignment. As more and more system-wide data are generated across different species, the concept of orthology-based meta-clustering demonstrated by OrthoClust can serve as a general computational framework for integration of other ‘omic’-scale data, such as protein-protein interactions.

## Results

### Cross-species modules in a multi-layer network

**Figure 1:**
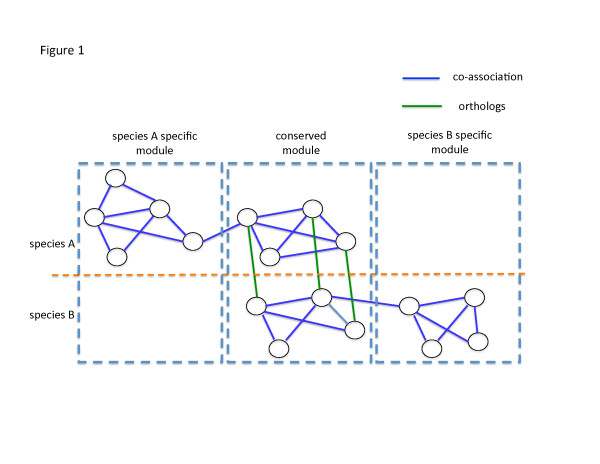
**An example to illustrate the idea of modules in a multi-layer network.** The co-association networks of species A and B are linked together to form a multi-layer network via orthologous relationship between genes. There are three modules. The middle one is a conserved module with genes from both species, corresponding to fundamental biological functions across different species. The left and right ones are specific modules consisting of genes from species A and B, respectively. They correspond to novel functions that emerged in each of the two species.

A co-association network is a representation of certain types of genomics data. The data can be rather simple, such as protein binding profiles, in which two genes are connected if their corresponding proteins can physically interact. In many cases, it can be high dimensional, such as genome-wide expression profiles. In this scenario, two genes are linked in a mathematically abstract way if their expression values across a variety of conditions are highly correlated. Despite the origin of the network, from a topological standpoint, a module is an interconnected region of the network where the density of edges is higher than the average density of the whole graph. Constituents of a module are presumably genes working in a coordinated fashion, that is, sharing a common function. We combined the co-association networks from different species to form a network with two types of edges representing two types of functional similarities. Mathematically this structure is a multi-layer network [13]. Genes in a species are connected if they are co-associated, whereas genes from different species are connected if they are orthologs. Figure 1 shows a simple example of such a multi-layer network. We extended the concept of modules used in co-association networks of individual species in a novel cross-species fashion. Here a module may comprise genes from multiple species, characterized by the two types of functional similarity in a cross-species manner. Within a module, from a molecular viewpoint, genes from the same species most likely share the same function as they are co-associated, co-expressed or physically bound together. Orthologs across different species (by definition homologs descended from the same ancestral gene), because of their sequence similarity, might have similar biological function from an evolutionary standpoint. Intuitively, a module should consist of nodes that form clique-like structures within a co-association network, as well as nodes that are linked by orthology relationships between layers of co-association networks. Nevertheless, as illustrated in Figure 1, it is entirely possible that a module in the multi-layer network consists of genes from a single species. In fact, this is the case when a novel function emerges for a particular species and the genes corresponding to the specific function do not have corresponding orthologs.

### Overview of OrthoClust

**Figure 2:**
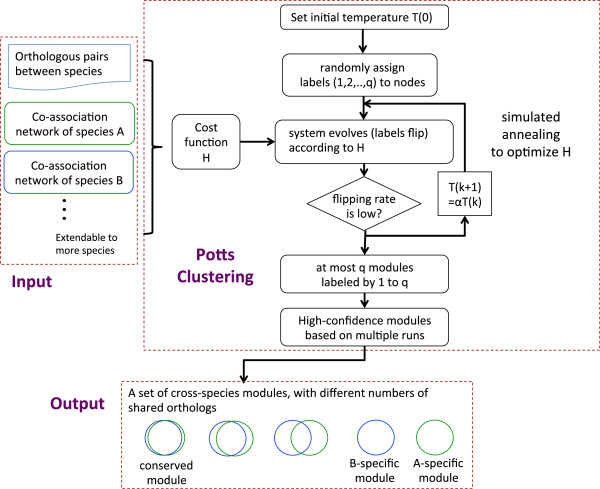
**Outline of OrthoClust.** The inputs of our pipeline are co-association networks from multiple species as well as orthology relationships. A cost function is defined based on the topology of the co-expression networks as well as orthology relationships. Each node can be in one of *q* possible states labeled by 1 to *q*. The cost function *H* is optimized by simulated annealing. In simulated annealing, labels are randomly assigned initially and are allowed to flip based on *H*. The temperature of the system is gradually lowered with a cooling factor α = 0.9. The algorithm stops if the flipping rate is low enough. The labels of nodes at the optimal configuration represent the assignment of nodes to modules. The algorithm is repeated multiple times. The resultant modules, represented by a set of Venn diagrams, could be specific or conserved.

Figure 2 shows the three major steps of OrthoClust: construction of the multi-layer network, defining the cost function of the system and assigning nodes to modules by multiple runs of simulated annealing.

#### Construction of the multi-layer network

The inputs of OrthoClust are the co-association networks of two or more species, and the orthology relationships between genes of the species of interest. Of course, co-association networks are derived from raw data, and there are various ways to arrive at the networks depending on the specific data and biological purpose. OrthoClust combines individual layers of co-association networks by connecting genes in different species via their orthology relationships. To account for the fact that many orthologous pairs are not one-to-one but many-to-many, orthologous links are weighted such that the weights are normalized by the number of orthologs of each node (Materials and methods).

#### Defining the cost function in the multi-layer network

OrthoClust defines a cost function in order to detect modules in a multi-layer network. Specifically, every node can take a discrete label σ ranging from 1 to *q*. Nodes with the same label will be assigned to the same module. *q* is therefore a parameter chosen to be the maximum number of modules allowed in the system. If the network has *M* nodes, there will be *M*^*q*^ ways (configurations) to assign nodes to modules. In general, OrthoClust can work for *N* species. For the case *N* = 2, each configuration is characterized by a cost function *H* defined as:


Here, S_1_ and S_2_ are the sets of genes for the two species, respectively. Λ_*ij*_ = *A*_*ij*_ - *k*_*i*_*k*_*j*_/2 *m*, with *k*_*i*_ = ∑ _*j*_*A*_*ij*_ and *m* = ∑ _*i*_*k*_*i*_*/2*. As *A* is a network adjacency matrix, the subtracted term is the expected number of links between nodes *i* and *j* in an ensemble of random graphs with the same degree distribution [14, 15]. Its presence in *H* is to reduce the contribution of links between nodes with higher degree (that is, hubs). The superscripts (1 or 2) correspond to the networks of two species. The value of the Kronecker delta  equals one if nodes *i* and *j* have the same label and zero otherwise. The first two terms of the cost function *H* are essentially the modularity functions of two individual networks [16]. In the standard modularity function, a network with high modularity means there is a high number of links between nodes in the same module, and a low number of links between nodes in different modules. The novelty of OrthoClust is the last term regarding the orthologous links between nodes in different layers of the co-association networks. It sums over O(*S*_1_, *S*_2_), that is, all the orthologous pairs between S_1_ and S_2_. As mentioned above, each pair of orthologs is weighted by *w*_*ij*_ to take into account the many-to-many orthology relationships (Materials and methods). Configurations in which orthologs have the same label will lower the cost function. The relative contribution between co-association edges and orthologous edges is controlled by a coupling constant *κ* (for determination of the constant, see below). In the language of statistical physics, the entire framework can be interpreted as a spin system called a *q*-state Potts model [17], which is a generalization of the Ising model. The cost function characterizes the energy of the spin (label) system and the optimal assignment of nodes to different modules is equivalent to the ground state of the Potts model.

#### Assigning nodes to modules by multiple runs of simulated annealing

To optimize the cost function, OrthoClust employs a standard simulated annealing procedure similar to the one used in [18]. Labels are randomly assigned initially, and updated via a heat bath algorithm. The temperature of the system is gradually lowered until the flipping rate of labels is lower than a certain threshold (Materials and methods). Although the labels have divided nodes of the network into modules, we do not directly use the resultant configuration due to the probabilistic nature of simulated annealing, but perform the annealing process R times. By summarizing the results using a co-appearance matrix (a matrix whose elements (*i,j*) represents how often the two nodes *i* and *j* co-appear in the same module), OrthoClust arrives at a set of modules by thresholding the co-appearance frequency and looking for nodes that co-appear often (Materials and methods). Often the sizes of the modules follow a power law distribution; tiny modules are therefore neglected (Materials and methods). OrthoClust is generally not very sensitive to the value of *q*. This is because, even though the system starts with many different labels (a high value of *q*), the large range of states will coalesce into a few modules and only a few labels will remain to cover the appropriate number of modules as the system cools down. In other words, the exact value of *q* is not very important as long as *q* is chosen to be large.

### Using OrthoClust for integrating expression profiles across species

A particular application for OrthoClust is to cluster expression profiles across species. Since OrthoClust is a network framework, raw expression profiles should be transformed into individual co-expression networks. Many algorithms have been proposed for this purpose based on calculating the *N* by *N* Pearson correlation matrix [19–22]. For our application, we found that a rank-based algorithm in which each gene is connected to the top *d* genes with the highest (absolute) Pearson correlation works best for resolving modules [19] (Materials and methods). It is well known that co-expression networks in many different species are modular, meaning that a subset of genes (a module) have a specific function [5, 23–25]; therefore, it is interesting to explore how these modules emerge in a cross-species fashion. Like various co-association networks constructed by correlating high-dimensional data, a co-expression edge can be assigned to have either a positive (+1) or a negative sign (-1) based on the sign of the correlation coefficient between two genes. Since anti-correlated genes do not work together, it is instructive to separate them into two different modules. This can be achieved by modifying the original cost function to separate the sets of positive and negative links in each species as specified by the superscripts (+ or -), that is:


The minus sign in front of the negative links means the effects of the negative links are opposite to those of the positive links, meaning that, in the favorable configurations, nodes in the same module are likely to be connected by positive links and nodes from different modules tend to be connected by negative links [26].

### Simultaneous clustering of expression profiles in worm and fly via OrthoClust

**Figure 3:**
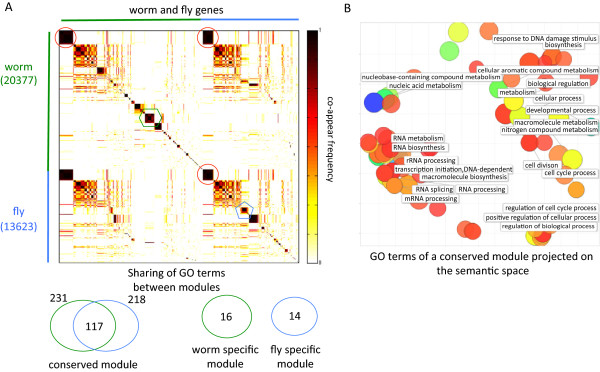
**Worm-fly modules resulted from OrthoClust. (A)** The co-appearance matrix of worm and fly genes. The worm and fly genes were sorted separately. Blocks along the diagonal are modules of worm and fly. Some blocks along the diagonal have strong co-appearance at the off-diagonal positions (see red circles as an example). These are conserved modules across worm and fly. In such modules, the corresponding worm and fly genes show strong overlap of Gene Ontology (GO) terms (*P* = 3.3 × 10^-16^, hypergeometric test). There are blocks along the diagonal that have no overlap at the off-diagonal positions (the blue pentagon and the green hexagon). They are the worm-specific and fly-specific modules. Such modules have rare overlap in terms of their GO terms (*P* = 0.035, hypergeometric test). **(B)** Enriched GO terms of a conserved module in worm and fly. Each circle represents a GO term, and the color code stands for statistical significance. The terms project onto a semantic space in which the geometric distance between GO terms mirrors their sematic distance. GO terms with similar meanings are packed together. GO terms correspond to fundamental functions like RNA biology, cell cycle, and so on.

As a demonstration, we applied OrthoClust to the transcriptomes of worm and fly generated by the modENCODE consortium [26]. In this analysis, the initial number of spin states *q* was chosen to be 250. We summarized the results for R = 32 annealing runs (more details in the section 'Robustness analysis' below) using a *M*-by-*M* co-appearance matrix, where *M* is the size of the multi-layer network (the total number of genes in worm (20,377) plus fly (13,623) in this case). As shown in Figure 3A, there are blocks of worm and fly genes along the diagonal. These blocks consist of genes that co-appear often in various runs of annealing, representing different worm and fly modules. Of particular interests are the blocks of worm and fly genes that co-appear with high frequency in the off-diagonal positions. For instance, as highlighted in Figure 3A, a block of worm genes and a block of fly genes form a conserved module. As expected, they share a significant fraction of Gene Ontology (GO) terms (*P* = 3.3 × 10^-16^, hypergeometric test). Figure 3B shows the common GO terms between a set of worm genes and a set of fly genes in the conserved module. Most of the common GO terms refer to fundamental biological functions like RNA processing and cell cycles processes. On the other hand, blocks that do not overlap in the off-diagonal positions correspond to specific worm and fly modules. For instance, GO terms related to chitin (main component of exoskeletons of arthropods) activities were found in certain fly-specific modules. At a global level, we found that the size of modules follows a power-law distribution with an exponent of -1.89 (Figure S1 in Additional file 1). The power-law distribution observed includes certain large modules. Practically, one could implement extra steps to break down these large modules recursively.

### Separation of modules in the Gene Ontology space

**Figure 4:**
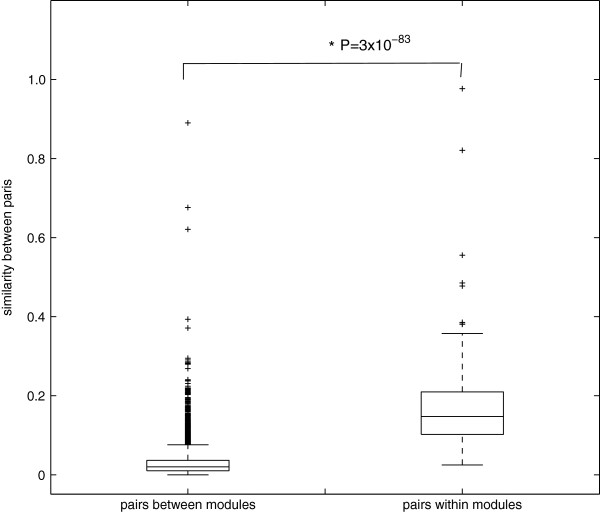
**The similarity of gene pairs within modules versus the similarity of gene pairs between modules.** Genes within modules are significantly more similar than genes from different modules.

As OrthoClust divides genes into modules based on how they are separated topologically in the multi-layer network, it is instructive to examine systematically how these modules are separated in functional space as defined by GO terms. To do so, we employed a metric to quantify the semantic similarity between all worm and fly genes (both intra-species and inter-species) based on the overlap of GO terms in a vector space model [28]. As shown in Figure 4, for the 150 modules obtained by clustering all worm and fly genes, the overlap between genes within a module is much higher than the overlap between genes across modules (*P* = 3 × 10^-83^, Wilcoxon test). Nevertheless, since two orthologous genes tend to have very similar GO terms, we further investigated whether such a high level of overlap between genes within a module is merely the consequence of orthology. We therefore repeated the analysis by removing all orthologs inside the modules. We found that the overlap between genes within modules is still significantly higher than the overlap across modules (*P* = 1.5 × 10^-45^, Wilcoxon test; Figure S2 in Additional file 1). Thus, we conclude that, in terms of GO annotation, OrthoClust has separated genes with different functions into different modules.

### Benchmarking modules based on co-regulation patterns

Apart from GO analysis in Figure 4, we further tested whether genes inside a module are indeed more functionally relevant by examining the number of common regulators they possess. We identified the binding targets of a set of worm and fly transcription factors based on ChIP-Seq experiments also generated by the modENCODE consortium [29] (Materials and methods). These ChIP-Seq experiments and the RNA-Seq experiments for expression profiles were performed in the same developmental stages. For all pairwise combinations of modules, we then counted the number of common transcription factors for each pair of genes (Figure S3 in Additional file 1). We found that pairs of genes within a module, on average, have more common transcription factors than pairs of genes in different modules (a 2.6-fold increase in worm and 1.6-fold increase in fly, *P* < 0.001 under permutation test). This result is consistent with general observations that a transcription factor tends to regulate targets sharing similar biological functions.

### Effects and the determination of the coupling constant κ

**Figure 5:**
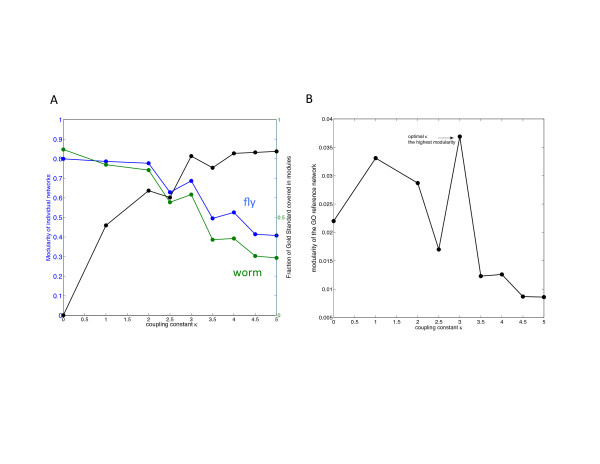
**Effects of κ. (A)** The effects of κ on clustering on the modularity of individual expression networks. As κ increases, the modularity scores of worm (green) and fly (blue) co-expression networks decrease. The fraction of metagenes whose components are found in the same modules decrease as κ increases. **(B)** The effects of κ on the modularity of the GO reference network. The modularity peaks at κ = 3, meaning that the modules defined by that particular value of coupling constant best separate the genes in terms of their GO annotations.

The cost function of OrthoClust takes into account two types of edges: co-association edges and orthology relationships. The coupling constant κ determines the relative contribution of the co-association links within a species and the orthologous links across species. A low value of κ means networks are likely to be clustered independently whereas a high value of κ means orthology links are more important and the label of a node tends to be aligned with its ortholog rather than its neighbors in the same network. In the clustering of gene expression profiles, we employed two independent methods to quantify the effects of tuning κ and determine its optimal value. First of all, we made use of a set of 1,288 metagenes obtained from [23] as our gold standard. These metagenes were constructed based on orthologs whose expression relationships are conserved across multiple species, including worm, fly and human. A metagene consists of genes from different genomes (worm and fly in this case) that presumably share the same function by considering their expression values across different conditions. Unlike our clustering approach, which is based on the optimization of a global cost function, metagenes were inferred by examining the likelihood that individual co-expression edges are transferred from one species to another in a local manner. This complementarity makes the set of metagenes a good gold standard for validation. Following our clustering framework, the constituents of a metagene should appear in the same module. As shown in Figure 5A, for a low value of κ, clustering was performed independently and it was rare that the worm and fly components of a metagene fall into the same module. Nevertheless, both the worm and fly networks have high modularity, meaning the two networks were independently well separated into modules. On the other hand, for a high value of κ, most of the metagenes satisfied the criterion whereas the resultant modularity of individual networks became low. The value of κ can be tuned so as to balance this tradeoff.

Another method we used to examine the effects of κ is the similarity measure between genes based on their GO annotation as described in the previous section. The similarity scores between each pair of the 34,000 worm and fly genes define a weighted network *W*, where the nodes are the genes and the edges are weighted by the pair-wise scores. Since the weighted network is not a multi-layer network but a single-layer network, its modularity can be quantified by a more traditional modularity function for weighted network defined as: , where *k*_*i*_ = ∑ _*j*_*W*_*ij*_, *m* = ∑ _*i*_*k*_*i*_*/2* and *σ*_*i*_ is the module label of node *i* [30]. A high modularity score means highly similar genes (in terms of GO annotation) are grouped in a module whereas distant genes are separated. In principle, this weighted network based on GO annotation serves as a benchmark for the multi-layer network defined by OrthoClust. A favorable way of assigning nodes to modules by OrthoClust therefore should also be a favorable way to divide the weighted network into modules. As shown in Figure 5B, for each value of κ, we found the way to assign nodes to the modules by OrthoClust and then calculated the corresponding modularity score of the weighted network. When the value of κ is too high or too low, the modularity score of the weighted network is low. The κ that maximizes the modularity score of the weighted network should therefore be the optimal κ for OrthoClust. Combing Figure 5A and Figure 5B, we picked κ = 3 as our optimal value.

### Weights associated with the orthology relationships

Orthology relationships between species connect layers of co-association networks. While the coupling parameter κ defines the overall relative contribution between intra-species and inter-species connections, the weight of each orthology connection could, in principle, be adjusted. It is very common that in eukaryotes many orthologs are many-to-many instead of one-to-one, mathematically forming various bipartite cliques in the multi-layer network. We tested OrthoClust by treating all the orthologous pairs equally in the cost function. We found that most of the cliques cannot be resolved, and their nodes got assigned to a single module (Figure S4 in Additional file 1). This implies the cost function favors very large cliques and is biased against the conserved clusters that are linked by one-to-one orthologs. To account for this effect, OrthoClust therefore weights down the orthologous link of a node by the number of orthologs it possesses. As shown in Figure S4 in Additional file 1, the weighted approach works better in resolving the huge cliques.

### Comparison with single-species clustering

**Figure 6:**
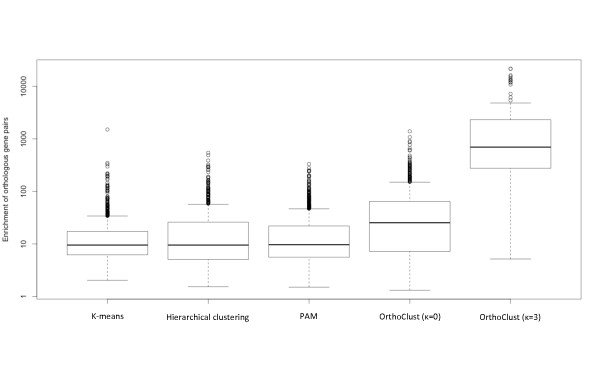
**Comparison between single-species clusters and cross-species clusters generated by OrthoClust.** The number of orthologous pairs for each pair of clusters generated by *K*-means, hierarchical clustering or PAM (Partition Around Medoids) is counted, and the fold enrichment over a null model is calculated (Materials and methods). Pairwise overlapping of clusters generated by single-species clustering (including OrthoClust with κ = 0) results in little enrichment of orthologous pairs compared with cross-species modules generated by OrthoClust.

The aim of OrthoClust is to perform clustering across multiple species in an integrated fashion. Naively, one could construct a cross-species module by performing clustering on individual species separately and concatenate the modules of different species by the orthologs they share. To examine this alternative approach, we performed single species clustering on the expression profiles of worm and fly separately using various standard methods (Materials and methods). We then tested for each combination of worm and fly modules, whether or not there is an enrichment of orthologs between them based on a simple hypergeometric test (Materials and methods). We found that even though there are certain combined worm-fly modules with significant enrichment of orthologous gene pairs, the enrichment is lower than the cross-species constructed by OrthoClust (Figure 6). This is of course not surprising because OrthoClust takes into account the orthology relationships in the algorithm. Nevertheless, this analysis suggested that, by using merely the co-expression data, it is in general less effective in finding the corresponding sets of genes in two species responsible for the same function. To show the result is not a consequence of the particular mathematical form of the cost function imposed by OrthoClust, we ran OrthoClust with κ = 0. As there was no coupling between two species in the cost function, the resultant sets of worm fly modules were essentially clustered independently. Again, we found that the combined worm-fly modules have lower enrichment of orthologous pairs compared with the case with optimal κ = 3. Interestingly, this analysis also manifests how the coupling term in the cost function brings two sets of independent modules closer together in terms of the sharing of orthologs.

### Comparison with network alignment

The concatenation of networks using orthology relationships resembles the problem of cross-species network alignment [12]. To compare OrthoClust with network alignment, we applied IsoRank [12] to align the worm and fly co-expression networks (Materials and methods). Again, using the metagenes obtained from [23] as a gold-standard, we found that 88% of metagenes were aligned by IsoRank (Figure S5 in Additional file 1), compared with 81% by OrthoClust. Although IsoRank slightly outperformed OrthoClust in identifying the corresponding functional genes between two species, it does not immediately report how these pairs form clusters. Motivated by [31], we looked for co-expression edges in two networks whose nodes are aligned by IsoRank. By connecting such edges in the network, we generated aligned subgraphs that could potentially be interpreted as modules conserved across two species. Among the gene-pairs that are predicted to be in the same module, we found that 43% are consistent with OrthoClust. The percentage is probably reasonable because aligned subgraphs do not really possess the properties of clusters signified by the dense connections between genes within a species.

### Robustness analysis

Simulated annealing was employed to optimize the cost function defined by OrthoClust. To reduce the effects of the stochastic nature of simulated annealing, we constructed the co-appearance matrix by repeating the annealing process R times. To determine R, we ran independent trials of R runs, resulting at different co-appearance matrices and thus different sets of clusters. We then compared the consistency between two sets of clusters by considering if two genes have been assigned to the same module by trial 1, whether or not they are assigned to a common module in trial 2. This is essentially done by calculating a confusion matrix (Materials and methods). By pairwise comparison of independent trials, we found that the overlap between trials increases as R increases (Figure S6 in Additional file 1). More specifically, the overlap increases from 46% for R = 8 to 65% for R = 32, and 75% for R = 64. The statistically significant results for R = 32 in the previous analysis show that the value offers a reasonable compromise between computational cost and robustness. We then further superposed different trials to construct a co-appearance matrix with 128 runs, and thus a set of 'most accurate' clusters. We then calculated the consistency between the ultimate set with sets constructed with smaller values of R (Figure S6 in Additional file 1). We found that the average consistency between clusters generated with R = 32 and the ultimate set is 76%.

### Mapping uncharacterized elements to modules

**Figure 7:**
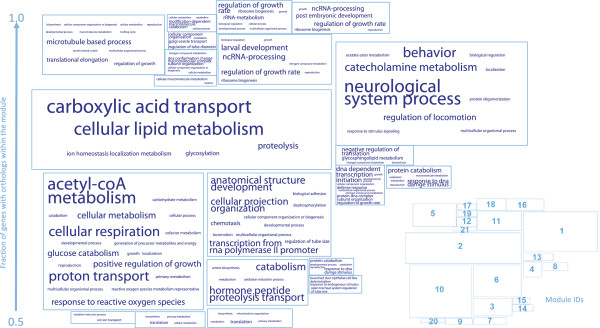
**The set of conserved worm-fly modules and their annotated functions.** The boxes represent modules. For each module, potential functions are summarized by keywords associated with enriched GO terms in a tag cloud. The font size of a keyword is proportional to the frequency of occurrence of the corresponding GO terms in the module. The position of a module in the vertical direction represents the fraction of genes with orthologous partners in the module.

Apart from understanding the modular nature of biological processes, clustering expression profiles is very useful for inferring the putative functions of uncharacterized proteins [32] as well as ncRNAs [33, 34]. The essence of this approach is 'guilt by association': if an uncharacterized element is highly co-expressed with a core set of genes, one can infer the function of the gene based on the functions of genes within the core set. Nevertheless, most core sets were constructed by single-species clustering. The cross-species modules constructed by OrthoClust can potentially serve as an anchor to relate uncharacterized but analogous elements from different species. To explore this avenue, we constructed modules using a set of core worm and fly genes (worm-fly orthologs) by OrthoClust (Materials and methods; Figure S7 in Additional file 1), arriving at a set of 21 core worm-fly modules with similar proportions of worm and fly genes (Additional file 2). We further investigated the functions of these modules based on their enriched GO terms (Materials and methods). For each module, by clustering the enriched GO terms, we assigned a list of representative keywords as their characteristic functions (Figure 7). For instance, module 1 is signified by neurological system process and module 2 by cellular lipid metabolism. As expected, many genes in these modules have orthologous partners within the module. In 18 out of the 21 modules, the fraction of genes with orthologous partners is higher than 80%.

We then mapped worm and fly ncRNAs to the 21 modules based on their expression profiles (Materials and methods). Though there is no gold standard available to evaluate systematically the performance of the mapping, we found examples suggesting that ncRNAs from different species could be linked together in terms of their potential functions. For instance, sphinx, the fly long non-coding RNA (lncRNA) expressed in the brain, was shown to be involved in regulation of male courtship behavior [35]. In our analysis, this lncRNA was mapped to module 1, which is characterized by neurological system process and behavior. On the other hand, linc-10 and linc-104, worm large intergenic non-coding RNAs (lincRNAs) that are highly expressed in male-related stages [36], were mapped to the same module.

In addition to the mapping, we also found that some modules are enriched with different classes of ncRNAs (Figure S8 in Additional file 1). The list of worm and fly ncRNAs we tested and the modules they mapped to can be found in Additional file 3.

### Generalization to N species

OrthoClust is a general framework not only applicable to the clustering of expression profiles but in general other genomics data in the form of co-association networks. In addition, the framework can be readily applied to more than two species by modifying the cost function. In general, for *N* species, the cost function will have *N* terms for the co-association networks, and *N*(*N* - 1)/2 terms for the orthologs between all pairs of species. For instance, if *N* = 3, the cost function can be written as:


Here, *S*_*1*_*, S*_*2*_*, S*_*3*_ stand for three different species. The inner summation is the modularity function for the network of a single species. The outer summation sums the three networks together. The extra terms represent the coupling (with coupling constant κ) between three pairwise combinations, namely the orthologous pairs represented by *O(*S_*1*_*, S*_*2*_*)*, *O*(*S*_1_, *S*_3_) and *O*(*S*_2_, *S*_3_). The coupling constant κ can be determined by the same approach we explained in the example of two species. Of particular interest is the third-order coupling term for the 1-1-1 triplets, *O*(*S*_1_, *S*_2_, *S*_3_). A triplet consists of three genes from three species that are orthologous to one another in a one-to-one fashion; that is, for triplet , apart from , no other gene in *S*_*1*_ is orthologous to  and vice versa. In this cost function*,* the third-order term favors a 1-1-1 triplet to have the same label. The 1-1-1 triplets are of particular importance among all the orthologous triplets because they correspond to particularly conserved biological function. Genes performing less conserved functions are more likely to undergo gene duplication and end up with many-to-many orthologs.

## Discussion and conclusions

In this study, we present OrthoClust, an orthology-based network framework that performs data clustering across multiple species. Due to the rapid increase of data from many species, a novel meta-clustering framework that integrates data from different species will be highly useful for comparative genomics. In OrthoClust, a module is defined based on evolutionary information as well as co-association information. A conserved module groups genes from multiple species corresponding to a common biological function, whereas a species-specific module consists of genes that are responsible for a specific function novel to a species. Though we have focused on expression data for illustration, OrthoClust can be readily applied to other high-dimensional data like histone modification patterns or protein-protein interactions by appropriately modifying the cost function. For instance, in the context of interactome, OrthoClust can be used to detect modules in protein-protein interaction networks in a cross-species context and examine the evolution of protein complexes.

In single-species clustering, a conserved gene can be grouped in a species-specific module simply because of their strong tie. By incorporating orthology relationships between species, OrthoClust detects better the conserved modules. The concept of cross-species modules complements the principle of 'guilt by association' because it may potentially lead to functionally analogous elements across species. This is of fundamental interest for elements like ncRNAs because only short regions of ncRNAs are constrained by structure or sequence-specific interactions and, compared to protein-coding genes, this discrepancy in selection pressure makes it very hard to find orthologous ncRNAs by their sequence [37]. RNA-Seq experiments have found that over 60% of the genome is transcribed, and many transcriptionally active regions (TARs) have been identified [38]. Therefore, mapping onto OrthoClust modules based on expression profiles serves at least as an intermediate step to inferring the putative functions of the vast amount of transcriptionally active regions.

Like many clustering algorithms, OrthoClust is unsupervised. Generalization to supervised clustering based on various gold standards are possible in principle. Nevertheless, the essence of OrthoClust is a global optimization process. The exact mathematical form of the cost function depends on the number of species and the types of data. In our application of OrthoClust to expression networks, we have separated the effects of positive and negative edges. The procedure is analogous to the idea of structural balance in the context of social networks [39]. Nevertheless, the cost function of such a signed network has a more complicated energy landscape and therefore makes the optimization process trickier. In this analysis, we did not find a significant improvement compared with the unsigned case. Another aspect concerning the mathematical form of the cost function is worth mentioning. As pointed out by several studies, finding network communities or modules using a global objective function is subject to resolution limits [40, 41]. The limit explains the existence of giant modules detected by OrthoClust. In principle, this could be complemented by a recursive scheme. Nevertheless, an extra coupling parameter λ can be added to the definition of Λ such that Λ_*ij*_ = *A*_*ij*_ - λ *k*_*i*_*k*_*j*_/2 *m*. The parameter can control the size of resultant modules [18] but it is commonly chosen to be 1 by convention. In principle, the coupling parameter can be tuned to obtain a better resolution, or to obtain sizes that are more biologically relevant. In terms of minimization of the cost function, we used simulated annealing as a conceptual demonstration. Though it is theoretically possible to obtain the optimal solution, it is computationally expensive. Indeed, there is no simple theory to address the convergence time, which may present problems especially for multiple species scenarios. In principle, one can replace simulated annealing with another heuristic [42]; there are other faster approaches for module identification for single-layered networks, for instance, spectral techniques [16]. A recent study combining various co-expression networks from the same species based on tensor computation point in a similar direction [43]. As simulated annealing does not scale very well as the number of species increases, the other approaches described may present more efficient solutions.

Efforts have been spent on the comparison of networks from different species (for example, [12, 44–47]). Earlier work focused on whether individual interactions are conserved across species [44], and, more recently, attempts have been made to quantify the rate of rewiring [47]. In particular, network alignment is a well-established analysis for cross-species network comparison [44–46]. Though OrthoClust resembles network alignment, the two problems are quite different. The essence of network alignment is to understand how individual nodes and edges in one network have their counterparts in another network, whereas OrthoClust focuses on whether genes working together in one species (signified by the dense connections between genes) would preserve the collaboration in another species (another dense region). Network alignment thus involves greater topological details, and to a certain extent is a harder problem. As many of the networks constructed are rather incomplete and there are possibly false positives, detecting modules is generally less sensitive to these errors compared with network alignment. It is worthwhile to point out that while the original motivation of some network alignment algorithms like IsoRank is to improve orthology prediction [12], conserved modules in OrthoClust could be interpreted as potential homologs and analogs that perform similar functions in different organisms. The incorporation of corresponding co-association networks is actually important because common orthology detection approaches focus on the sequence level [48]. Because of the resemblance of network alignment and cross-species clustering, one could also modify OrthoClust by replacing the orthology relationships using aligned gene-pairs. As OrthoClust is a flexible computational framework, such modification would be technically straightforward but conceptually interesting to explore.

## Materials and methods

### Datasets of transcriptome and orthologous pairs

Transcriptome profiling data from worm and fly were generated by the modENCODE consortia using RNA-Seq [27]. The expression values of worm and fly were measured across 33 and 30 developmental stages, respectively. The total 10,031 worm-fly orthologous pairs (including one-to-one, one-to-many, many-to-many relationships from 5,769 unique worm orthologous genes and from 5,507 unique fly orthologous genes) between worm and fly were downloaded from the modENCODE website as they were compiled by the consortium [27]. At the genome-wide level, there are 20,377 worm genes and 13,623 fly genes. For each species, expression values in different developmental stages or cell lines were log-transformed and standardized and Pearson correlation coefficients were calculated for each pair of genes. The list of ncRNAs in worm and fly were obtained from wormbase [49] and flybase [30], including lncRNAs (228 in worm, 852 in fly), microRNAs (211 in worm, 215 in fly), small nucleolar RNAs (snoRNAs) (141 in worm, 287 in fly) and tRNAs (236 in worm, 238 in fly); more than 100 RNAs of each type are present in both worm and fly. The accession numbers for raw data, the processed expression values from the RNA-Seq experiments, and the orthologous pairs between worm and fly can be found in Additional file 4.

### More details on OrthoClust

#### The cost function

To take into account the fact that many orthologous pairs are not one-to-one but many-to-many, the contribution of a pair of orthologs to the generalized modularity function is not 1, but normalized by the number of orthologs. For example, if gene *i* from species 1 is orthologous to  genes in species 2 including gene *j*’ whereas gene *j*’ in species 2 is orthologous to  genes in species 1 including gene *i*, the weight *w*_*ij’*_ is defined as . For simplicity, this modification is not displayed in the main equation.

#### Simulated annealing

Standard simulated annealing was employed. Spin values were randomly assigned initially, and updated via a heat bath algorithm. The initial temperature was chosen in a way such that the flipping rate (the probability that a node changes its spin state) was higher than 1 - 1/*q*. The temperature was gradually decreased with a cooling factor 0.9, until the flipping rate was less than 1%.

### More details on applying OrthoClust to cluster expression profiles

#### Construction of individual co-expression networks

Many algorithms have been proposed to transform raw expression profiles into individual co-expression networks based on calculating the N-by-N Pearson correlation matrix [19–22]. There are two classes of algorithms: those imposing a global threshold on the values of the correlation coefficients for all genes (value-based), and those that locally allow each gene to connect to the top *d* most correlated genes (rank-based). Networks constructed by global value-based algorithms were found to be more difficult to resolve into smaller modules. Therefore, a local rank-based algorithm in which each gene is connected to the top *d* genes with the highest (absolute) Pearson correlation was employed [19]. The value of *d* was chosen in order to keep the sparsity of networks. More specifically, *d* is the smallest value such that all genes from individual species independently form giant connected networks In general, if *d* is very small, the resultant network by definition cannot form a giant connected graph. On the other hand, if *d* is very large, the network would not be sparse. In the worm fly analysis, *d* was chosen to be 5. Even though the numbers of nodes and edges in the two co-expression networks vary, the average number of links per node is quite similar (6.29 for worm and 6.56 for fly).

#### Decomposition of modules in worm and fly

In the genome-wide worm fly analysis, a stringent threshold (0.95) for co-appearance was employed for the co-appearance matrix shown in Figure 3A. Nodes that ended up with the same spin value for more than 95% of trials were assigned to the same module. Tiny clusters were neglected, arriving at a set of about 150 modules covering about 80% of nodes. Proper GO terms were found in the remaining modules.

### GO similarity between pairs of genes

There are many metrics for quantifying gene functional similarity based on GO terms [28, 50–53]. The metric used in this study was adopted from [28]. The relationship where gene *i* is annotated with GO term *j* was represented by an adjacency matrix B, and further a matrix G was defined such that  where *n* is the number of genes. In matrix G, the contribution of a GO term *j* to a gene is weighted by its inverse document frequency, a quantity commonly used in text-mining [54]. High-level GO terms present in many genes were therefore weighted down. The similarity score between two genes *k*_*1*_ and *k*_*2*_ was defined as the cosine of the two corresponding vectors (the  row and the  row in the G matrix).

### Regulatory patterns of modules

ChIP-Seq data of 26 fly transcription factors and 79 worm transcription factors across various developmental stages (together 220 experiments in worm and 93 experiments in fly) were downloaded from the modENCODE consortium. For each ChIP-Seq experiment, binding targets of the transcription factors were identified by TIP [55] with a *q*-value cutoff of 0.01. The results of these experiments were superposed together to form the transcriptional regulatory networks for worm and fly (12,648 edges for worm and 1,187 edges for fly). The number of common transcription factors for a pair of genes was determined based on the resultant networks.

### Comparison with single-species clustering

Standard clustering procedures, including *k*-means, hierarchical clustering and PAM (Partition Around Medoids), were applied for transcriptome profiling data from worm and fly. Resultant modules of size less than five genes were neglected. In summary, for all three methods, about 200 worm modules and 200 fly modules remained. For each combination of these modules, the number of orthologous pairs between worm and fly genes was counted. The number of orthologous pairs was then compared to the expected number , where *n*_*w*_ and *n*_*f*_ are the number of genes in the worm and fly modules, *N*_*w*_ and *N*_*f*_ are the total number of worm and fly genes, and *O*_*wf*_ is the number of orthologous pairs between worm and fly. Only combinations with enrichment of orthologous pairs were kept for the display in Figure 6 (*P* < 0.05, hypergeometric test). For OrthoClust with κ = 0, modules of size less than 5 genes were also neglected in the comparison, resulting in 314 worm modules and 227 fly modules.

### Comparison with network alignment

We applied IsoRank to align the worm and fly co-expression networks. The sequence identity between pairs of worm and fly proteins were downloaded from [12]. We tuned the intrinsic parameter α but we did not find systematic trends. We then used α = 0.5 and looked for co-expression edges in two networks whose nodes are aligned. Disconnected components formed by these aligned edges were used as potential seeds of conserved modules because they consist of sets of worm genes and fly genes that are perfectly aligned.

### Robustness analysis

To compare two sets of clusters A and B, all possible N(N - 1)/2 pairs of genes were divided into four categories: (I) assigned to the same module by both A and B; (II) assigned to the same module by A but not by B; (III) assigned to the same module by B but not by A; (IV) assigned to different modules by both A and B. Because the number of pairs in category IV (true negative to a certain extent) is orders of magnitude higher than the others, the overlap between A and B was defined as I/(I + II + III). The number of pairs in category I can be viewed as the true positive. The method is motivated by [56].

### More details on inferring the functions of worm fly ncRNAs

#### Modules based on worm fly core set

OrthoClust was applied to the set of orthologs between worm and fly, consisting of 5,059 worm genes and 4,863 fly genes. The coupling constant was determined using the same scheme illustrated in the main text. A set of 21 modules that each had more than 15 genes and with enriched GO terms was derived. As expected, the similarity between genes within modules was higher than the similarity between genes across modules (*P* = 1 × 10^-83^, Wilcoxon test). To annotate the functions of a module, the enriched GO terms among genes were obtained using the tool REVIGO [57]. The enriched GO terms were clustered into groups labeled by representative keywords given by REVIGO. The list of keywords was displayed using tag clouds (using the software TagCrowd [58]) in which the size of a keyword is proportional to the number of GO terms in the group.

#### Mapping ncRNAs to modules

Using RNA-Seq data generated by the modENCODE consortium, the expression profiles of ncRNAs were calculated under the same set of developmental stages as for the protein-coding genes. Annotation of ncRNAs was based on the latest version of wormbase [49] and flybase [59]. The ncRNAs were then mapped to the 21 modules based on the correlation between expression levels. More specifically, the correlation between the expression of the ncRNAs and the expression of all protein-coding genes was calculated. A null distribution was constructed by randomly shuffling the expression values of the ncRNAs 10 times, calculating the correlation coefficients between the randomized expression profile with expression profiles of all the protein coding genes, and pooling all values together. A set of protein-coding neighbors of the ncRNAs was identified as the set of most correlated protein-coding genes with a false discovery rate of less than 5% with respect to the null distribution. The enrichment of the set of neighbors in every module was calculated by a hypergeometric test. The ncRNAs would be mapped to a given module if *P* < 0.01. An ncRNA could be mapped to multiple modules.

#### Enrichment of different classes of ncRNAs in modules

To obtain the enrichment of a particular class of ncRNAs (microRNAs, tRNAs, snoRNAs, lncRNAs) with respect to the set of all ncRNAs in a given module, a hypergeometric test was employed to calculate the significance of the fraction of mapped ncRNAs of this class to four classes in the module against the fraction of total mapped ncRNAs of this class to four classes across all modules.

### Software availability

The code used for optimizing cost function (in MATLAB and R) is available online [60].

## Electronic supplementary material

Additional file 1: **Figures S1 to S8.** (PDF 680 KB)

Additional file 2: **Dataset S1.** Worm and fly genes in the 21 core modules. (CSV 55 KB)

Additional file 3: **Dataset S2.** All worm and fly ncRNAs used in the analysis and modules they were mapped. The rows are ncRNAs. The columns are the modules. The values 1 and 0 indicate whether a ncRNA is mapped to a module or not. (CSV 75 KB)

Additional file 4: **Dataset S3.** All modENCODE RNA-Seq datasets used in our study. (XLSX 355 KB)

## Abbreviations

GO: Gene Ontology
lncRNA: long non-coding RNA
ncRNA: non-coding RNA
snoRNA: small nucleolar RNA.

## References

[1] Berger B, Peng J, Singh M (2013). Computational solutions for omics data. Nat Rev Genet.

[2] Soon WW, Hariharan M, Snyder MP (2013). High-throughput sequencing for biology and medicine. Mol Syst Biol.

[3] Alon U (2003). Biological networks: the tinkerer as an engineer. Science.

[4] Hartwell LH, Hopfield JJ, Leibler S, Murray AW (1999). From molecular to modular cell biology. Nature.

[5] Langfelder P, Horvath S (2008). WGCNA: an R package for weighted correlation network analysis. BMC Bioinformatics.

[6] Eisen MB, Spellman PT, Brown PO, Botstein D (1998). Cluster analysis and display of genome-wide expression patterns. Proc Natl Acad Sci U S A.

[7] Tamayo P, Slonim D, Mesirov J, Zhu Q, Kitareewan S, Dmitrovsky E, Lander ES, Golub TR (1999). Interpreting patterns of gene expression with self-organizing maps: Methods and application to hematopoietic differentiation. Proc Natl Acad Sci.

[8] Kluger Y, Basri R, Chang JT, Gerstein M (2003). Spectral biclustering of microarray data: coclustering genes and conditions. Genome Res.

[9] Agrawal H, Domany E (2003). Potts ferromagnets on coexpressed gene networks: identifying maximally stable partitions. Phys Rev Lett.

[10] Mortazavi A, Williams BA, McCue K, Schaeffer L, Wold B (2008). Mapping and quantifying mammalian transcriptomes by RNA-Seq. Nat Methods.

[11] Wang Z, Gerstein M, Snyder M (2009). RNA-Seq: a revolutionary tool for transcriptomics. Nat Rev Genet.

[12] Singh R, Xu J, Berger B (2008). Global alignment of multiple protein interaction networks with application to functional orthology detection. Proc Natl Acad Sci U S A.

[13] Mucha PJ, Richardson T, Macon K, Porter MA, Onnela J-P (2010). Community structure in time-dependent, multiscale, and multiplex networks. Science.

[14] Newman MEJ, Strogatz SH, Watts DJ (2001). Random graphs with arbitrary degree distributions and their applications. Phys Rev E.

[15] Maslov S, Sneppen K (2002). Specificity and stability in topology of protein networks. Science.

[16] Newman MEJ (2006). Modularity and community structure in networks. Proc Natl Acad Sci U S A.

[17] Wu FY (1982). The Potts model. Rev Mod Phys.

[18] Reichardt J, Bornholdt S (2004). Detecting fuzzy community structures in complex networks with a Potts model. Phys Rev Lett.

[19] Ruan J, Dean A, Zhang W (2010). A general co-expression network-based approach to gene expression analysis: comparison and applications. BMC Syst Biol.

[20] Zhou X, Kao M-CJ, Wong WH (2002). Transitive functional annotation by shortest-path analysis of gene expression data. Proc Natl Acad Sci U S A.

[21] Van Noort V, Snel B, Huynen MA (2004). The yeast coexpression network has a small-world, scale-free architecture and can be explained by a simple model. EMBO Rep.

[22] Jordan IK, Mariño-Ramírez L, Wolf YI, Koonin EV (2004). Conservation and coevolution in the scale-free human gene coexpression network. Mol Biol Evol.

[23] Stuart JM (2003). A gene-coexpression network for global discovery of conserved genetic modules. Science.

[24] Kang HJ, Kawasawa YI, Cheng F, Zhu Y, Xu X, Li M, Sousa AMM, Pletikos M, Meyer KA, Sedmak G, Guennel T, Shin Y, Johnson MB, Krsnik Ž, Mayer S, Fertuzinhos S, Umlauf S, Lisgo SN, Vortmeyer A, Weinberger DR, Mane S, Hyde TM, Huttner A, Reimers M, Kleinman JE, Šestan N (2011). Spatio-temporal transcriptome of the human brain. Nature.

[25] Mao L, Van Hemert JL, Dash S, Dickerson JA (2009). Arabidopsis gene co-expression network and its functional modules. BMC Bioinformatics.

[26] Traag VA, Bruggeman J (2009). Community detection in networks with positive and negative links. Phys Rev E.

[27] Gerstein MB, Rozowsky J, Yan K-K, Wang D, Cheng C, Brown JB, Davis CA, Hillier L, Sisu C, Li JJ, Pei B, Harmanci AO, Duff MO, Djebali S, Alexander RP, Alver B, Auerbach R, Bell K, Bickel PJ, Boeck ME, Boley NP, Booth BW, Cherbas L, Cherbas P, Di C, Dobin A, Drenkow J, Ewing B, Fang G, Fastuca M, *et al*.: **Comparative analysis of the transcriptome across distant species.** *Nature* doi:10.1038/nature13424 10.1038/nature13424 PMC4155737 25164755

[28] Chabalier J, Mosser J, Burgun A (2007). A transversal approach to predict gene product networks from ontology-based similarity. BMC Bioinformatics.

[29] Boyle AP, Araya CL, Brdlik C, Cayting P, Cheng C, Cheng Y, Gardner K, Hillier L, Janette J, Jiang L, Kasper D, Kawli T, Kheradpour P, Kundaje A, Li JJ, Ma L, Niu W, Rehm EJ, Rozowsky J, Slattery M, Spokony R, Terrell R, Vafeados D, Wang D, Weisdepp P, Wu Y-C, Xie D, Yan K-K, Feingold EA, Good PJ, *et al*.: **Comparative analysis of regulatory information and circuits across diverse species.** *Nature* doi:10.1038/nature13668 10.1038/nature13668 PMC4336544 25164757

[30] Newman MEJ (2004). Analysis of Weighted Networks. Phys Rev E.

[31] Ficklin SP, Feltus FA (2011). Gene coexpression network alignment and conservation of gene modules between two grass species: maize and rice. Plant Physiol.

[32] Oliver S (2000). Proteomics: Guilt-by-association goes global. Nature.

[33] Guttman M, Amit I, Garber M, French C, Lin MF, Feldser D, Huarte M, Zuk O, Carey BW, Cassady JP, Cabili MN, Jaenisch R, Mikkelsen TS, Jacks T, Hacohen N, Bernstein BE, Kellis M, Regev A, Rinn JL, Lander ES (2009). Chromatin signature reveals over a thousand highly conserved large non-coding RNAs in mammals. Nature.

[34] Liao Q, Liu C, Yuan X, Kang S, Miao R, Xiao H, Zhao G, Luo H, Bu D, Zhao H, Skogerbø G, Wu Z, Zhao Y (2011). Large-scale prediction of long non-coding RNA functions in a coding–non-coding gene co-expression network. Nucleic Acids Res.

[35] Dai H, Chen Y, Chen S, Mao Q, Kennedy D, Landback P, Eyre-Walker A, Du W, Long M (2008). The evolution of courtship behaviors through the origination of a new gene in Drosophila. Proc Natl Acad Sci U S A.

[36] Nam J-W, Bartel DP (2012). Long noncoding RNAs in C. elegans. Genome Res.

[37] Pang KC, Frith MC, Mattick JS (2006). Rapid evolution of noncoding RNAs: lack of conservation does not mean lack of function. Trends Genet.

[38] Djebali S, Davis CA, Merkel A, Dobin A, Lassmann T, Mortazavi A, Tanzer A, Lagarde J, Lin W, Schlesinger F, Xue C, Marinov GK, Khatun J, Williams BA, Zaleski C, Rozowsky J, Röder M, Kokocinski F, Abdelhamid RF, Alioto T, Antoshechkin I, Baer MT, Bar NS, Batut P, Bell K, Bell I, Chakrabortty S, Chen X, Chrast J, Curado J (2012). Landscape of transcription in human cells. Nature.

[39] Doreian P, Mrvar A (1996). A partitioning approach to structural balance. Soc Netw.

[40] Fortunato S, Barthélemy M (2007). Resolution limit in community detection. Proc Natl Acad Sci U S A.

[41] Kumpula JM, Saramäki J, Kaski K, Kertész J (2007). Limited resolution in complex network community detection with Potts model approach. Eur Phys J B Condens Matter Complex Syst.

[42] Blondel VD, Guillaume J-L, Lambiotte R, Lefebvre E (2008). Fast unfolding of communities in large networks. J Stat Mech Theory Exp.

[43] Li W, Liu C-C, Zhang T, Li H, Waterman MS, Zhou XJ (2011). Integrative analysis of many weighted Co-expression networks using tensor computation. PLoS Comput Biol.

[44] Yu H, Luscombe NM, Lu HX, Zhu X, Xia Y, Han J-DJ, Bertin N, Chung S, Vidal M, Gerstein M (2004). Annotation transfer between genomes: protein–protein interologs and protein–DNA regulogs. Genome Res.

[45] Berg J, Lassig M (2006). Cross-species analysis of biological networks by Bayesian alignment. Proc Natl Acad Sci U S A.

[46] Kelley BP, Sharan R, Karp RM, Sittler T, Root DE, Stockwell BR, Ideker T (2003). Conserved pathways within bacteria and yeast as revealed by global protein network alignment. Proc Natl Acad Sci U S A.

[47] Shou C, Bhardwaj N, Lam HYK, Yan K-K, Kim PM, Snyder M, Gerstein MB (2011). Measuring the evolutionary rewiring of biological networks. PLoS Comput Biol.

[48] Fang G, Bhardwaj N, Robilotto R, Gerstein MB (2010). Getting started in gene orthology and functional analysis. PLoS Comput Biol.

[49] Harris TW, Antoshechkin I, Bieri T, Blasiar D, Chan J, Chen WJ, De La Cruz N, Davis P, Duesbury M, Fang R, Fernandes J, Han M, Kishore R, Lee R, Müller H-M, Nakamura C, Ozersky P, Petcherski A, Rangarajan A, Rogers A, Schindelman G, Schwarz EM, Tuli MA, Van Auken K, Wang D, Wang X, Williams G, Yook K, Durbin R, Stein LD (2010). WormBase: a comprehensive resource for nematode research. Nucleic Acids Res.

[50] Lord PW, Stevens RD, Brass A, Goble CA (2003). Investigating semantic similarity measures across the Gene Ontology: the relationship between sequence and annotation. Bioinforma Oxf Engl.

[51] Huang DW, Sherman BT, Tan Q, Collins JR, Alvord WG, Roayaei J, Stephens R, Baseler MW, Lane HC, Lempicki RA (2007). The DAVID Gene Functional Classification Tool: a novel biological module-centric algorithm to functionally analyze large gene lists. Genome Biol.

[52] Mistry M, Pavlidis P (2008). Gene Ontology term overlap as a measure of gene functional similarity. BMC Bioinformatics.

[53] Yu H, Jansen R, Stolovitzky G, Gerstein M (2007). Total ancestry measure: quantifying the similarity in tree-like classification, with genomic applications. Bioinforma Oxf Engl.

[54] Jones KS (1972). A statistical interpretation of term specificity and its application in retrieval. J Doc.

[55] Cheng C, Min R, Gerstein M (2011). TIP: A probabilistic method for identifying transcription factor target genes from ChIP-seq binding profiles. Bioinformatics.

[56] Brohée S, van Helden J (2006). Evaluation of clustering algorithms for protein-protein interaction networks. BMC Bioinformatics.

[57] Supek F, Bošnjak M, Škunca N, Šmuc T (2011). REVIGO summarizes and visualizes long lists of gene ontology terms. PLoS One.

[58] **TagCrowd** [http://tagcrowd.com/]

[59] Marygold SJ, Leyland PC, Seal RL, Goodman JL, Thurmond J, Strelets VB, Wilson RJ, FlyBase consortium (2013). FlyBase: improvements to the bibliography. Nucleic Acids Res.

[60] **OrthoClust** [https://github.com/gersteinlab/OrthoClust]

