# Supplementary material for: OrthoClust: an orthology-based network framework for clustering data across multiple species
Source: Genome Biol. 2014 Aug 28;15(8):R100. doi: 10.1186/gb-2014-15-8-r100 (PMC4289247; doi:10.1186/gb-2014-15-8-r100)
Supplement: Supplementary file 1 — Additional file 1: Figures S1 to S8. (PDF 680 KB) [file 13059_2014_3371_MOESM1_ESM.pdf]

OrthoClust: An orthology-based network framework for expression clustering across multiple species

Supplementary Figure 1

Supplementary Figure 2

Supplementary Figure 3

Supplementary Figure 4

Supplementary Figure 5

Supplementary Figure 6

Supplementary Figure 7

Supplementary Figure 8

Supplementary Figure 1

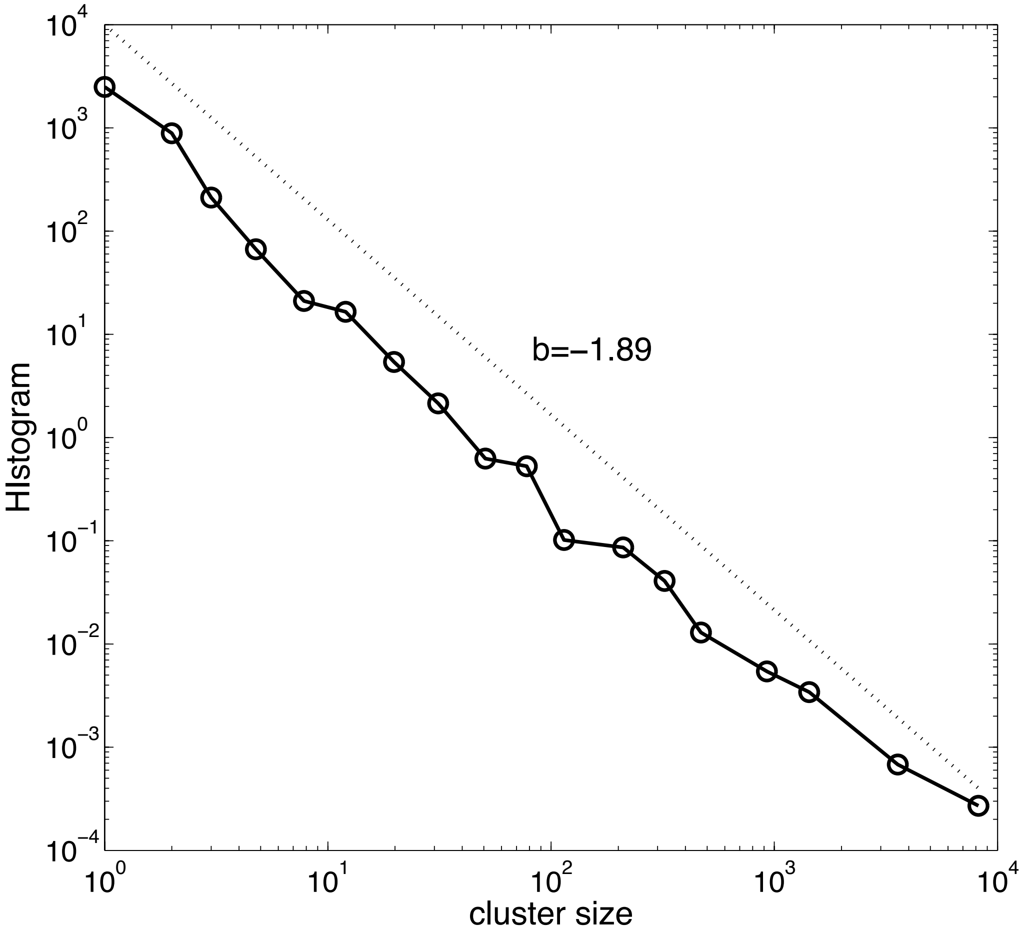

The size distribution of the resultant modules. The sizes follow a power law distribution of exponent -1.89.

Supplementary Figure 2

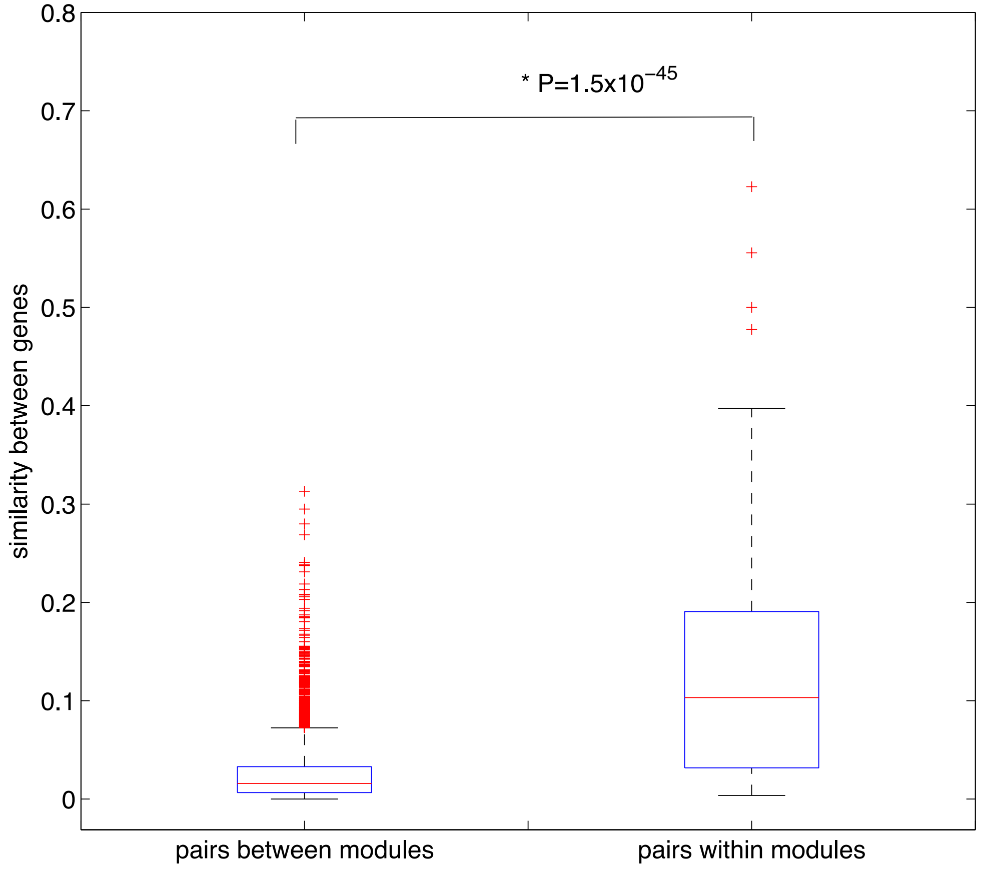

The similarity between genes within modules versus the similarity between genes between modules. Unlike Figure 4 in the main text, the orthologs inside modules were removed. The overlap between genes within modules is still significantly higher.

Supplementary Figure 3

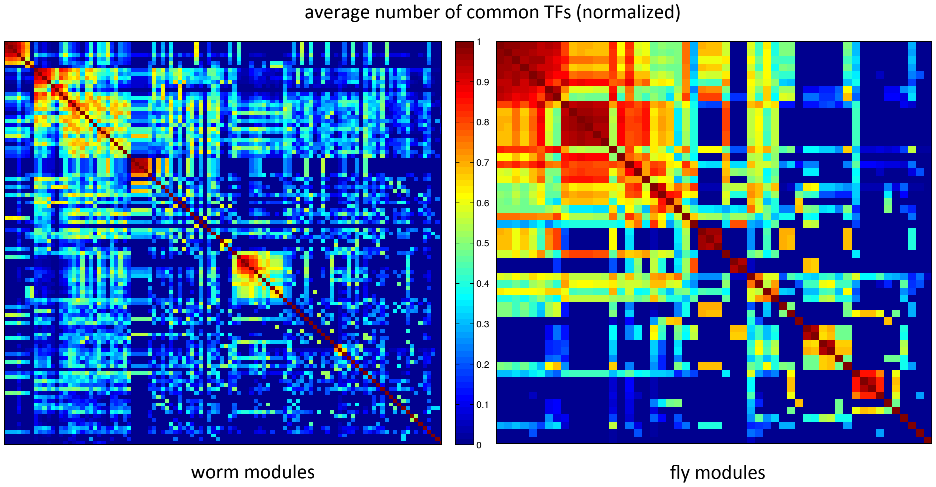

Average number of common transcription factors for pairs of genes within modules and between modules. Matrix element (i,j) means for all pairwise combinations of genes between modules i and module j, the average number of common transcription factors. The high average along diagonal means genes within a module share more common regulators than genes between modules. For visualization, the elements are scaled such that $\frac{M(i,j)}{\sqrt{M(i,i)M(j,j)}} \rightarrow M(i,j)$.

Supplementary Figure 4

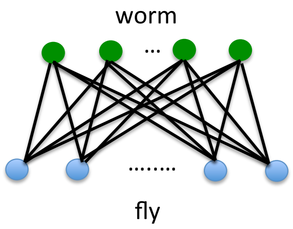

Number of worm genes	Number of fly genes	Number of modules by unweighted clustering	Number of modules by weighted clustering
67	22	1	68
20	43	1	31
8	25	1	17
5	23	1	6
20	8	1	23
18	12	1	16
17	17	1	2
8	17	1	22
17	2	7	17
13	10	1	4

Clustering of the top bipartite cliques (many-to-many orthologous pairs between worm and fly) using unweighted versus weighted orthologous links. Unweighted approach cannot resolve the cliques into different clusters.

Supplementary Figure 5

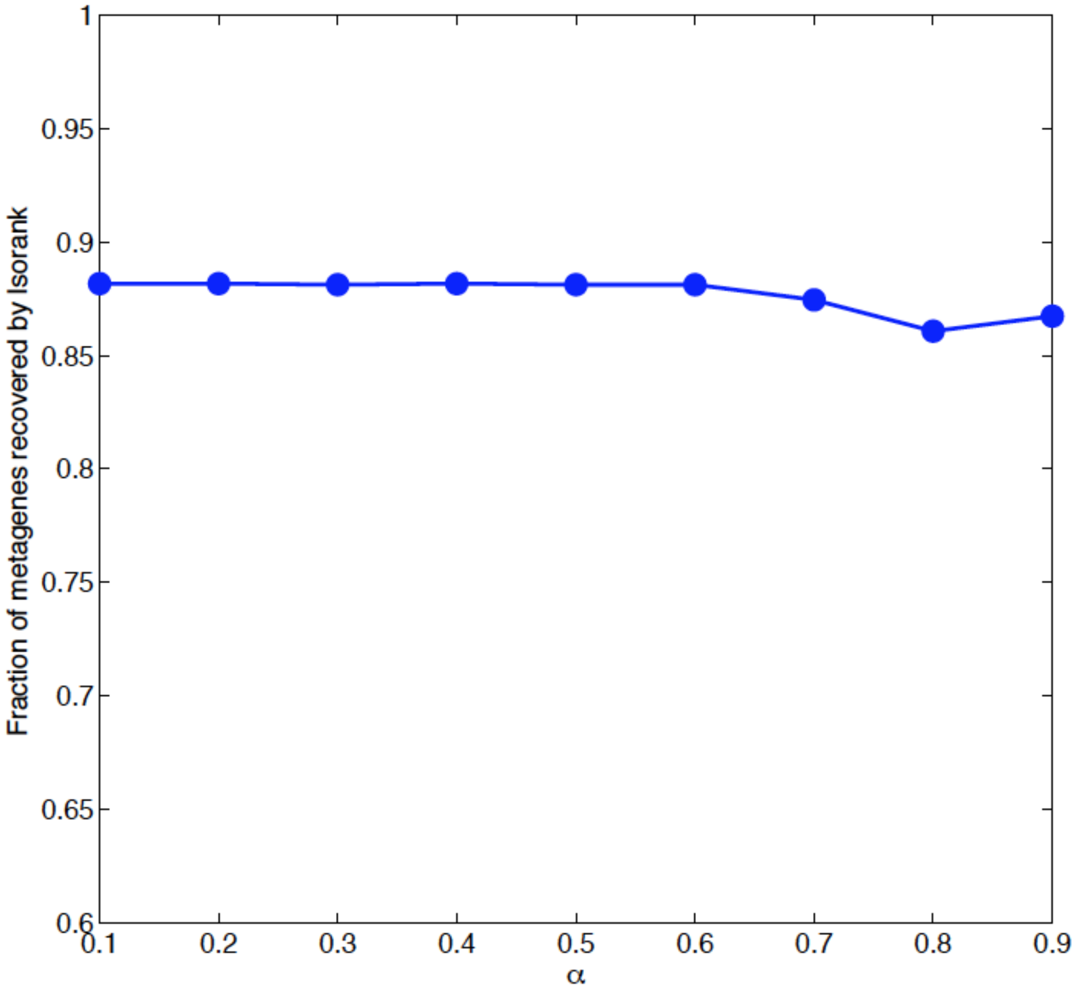

Fraction of metagenes recovered by IsoRank with respect to the intrinsic parameter α.

Supplementary Figure 6

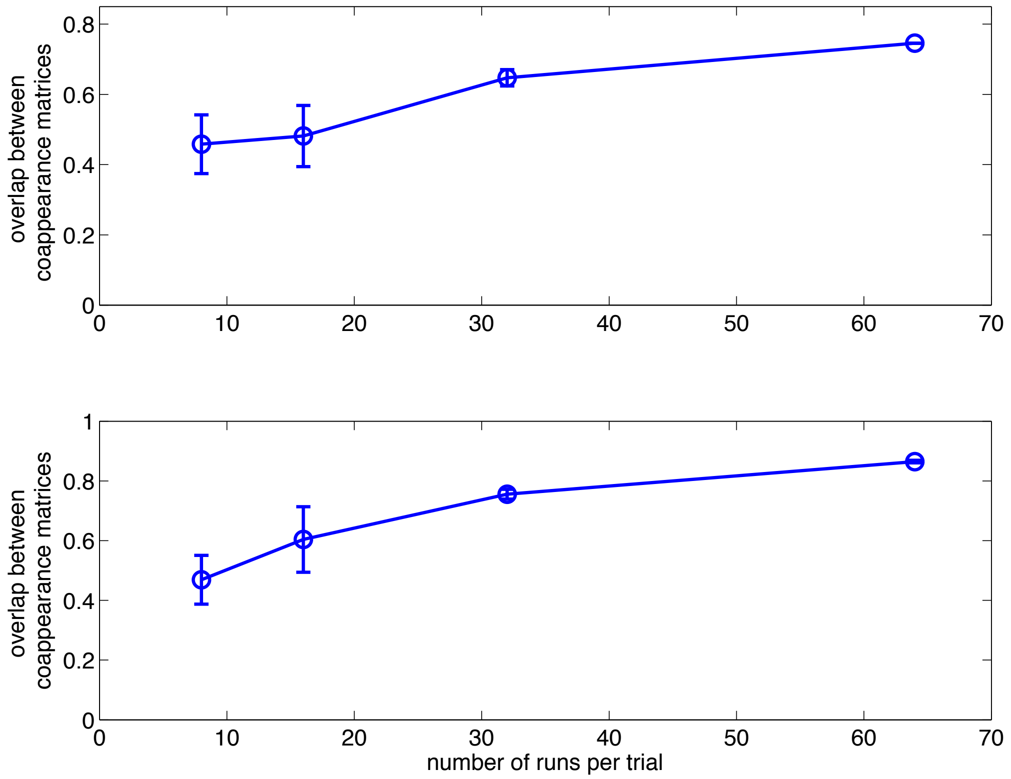

Overlap between sets of clusters. A set of clusters (a trial) is constructed by performing simulated annealing R times. Upper panel: average pairwise overlap between clusters constructed by the same number of trials. Lower panel: pairwise overlap between clusters constructed trials of R runs and trial with 128 runs.

Supplementary Figure 7

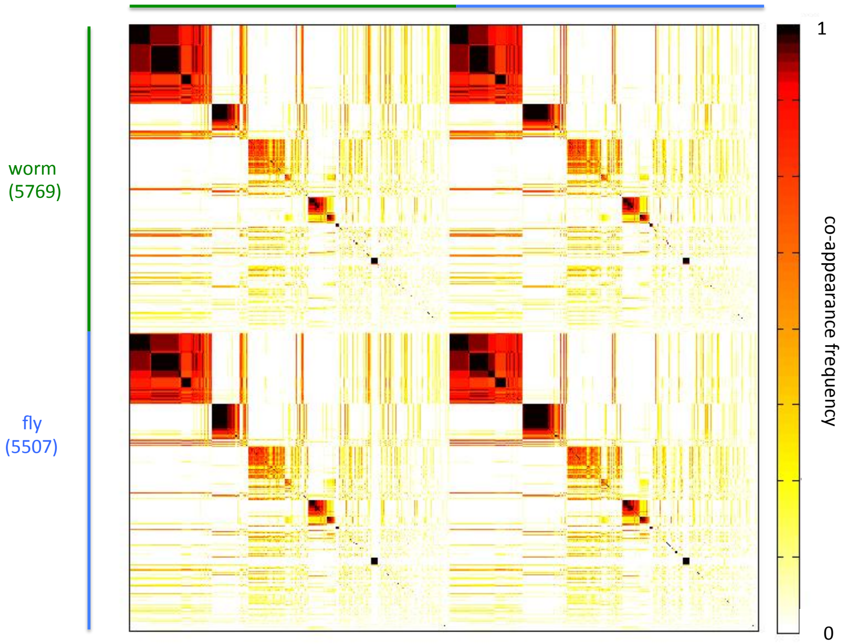

The co-appearance matrix for worm-fly orthologs. Unlike the genome-wide clustering, all the modules are conserved in nature, as evident from the block structures in the off-diagonal positions.

Supplementary Figure 8

Figure S9

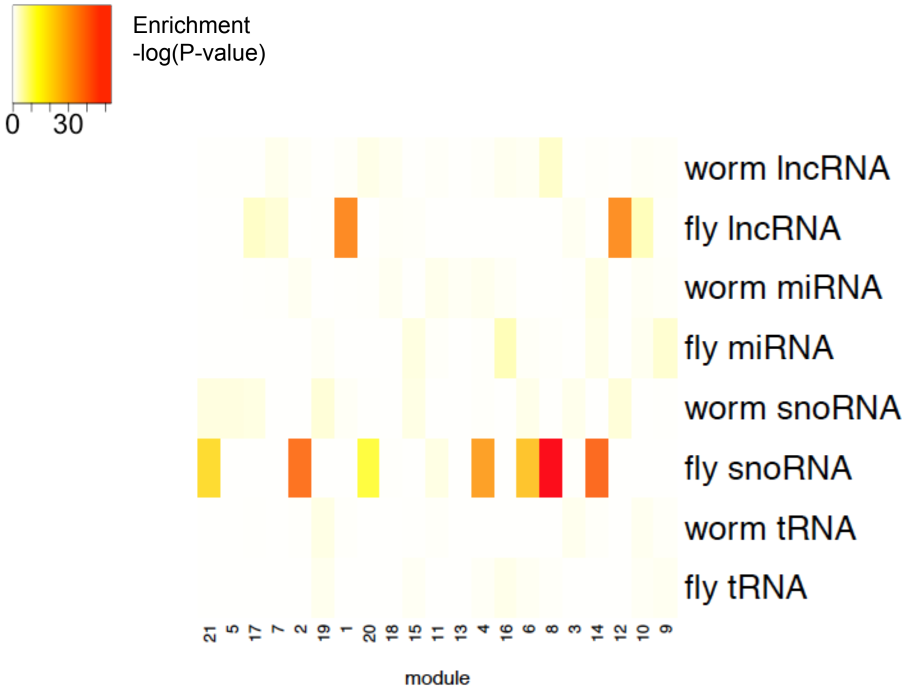

Enrichment of different classes of ncRNAs in modules. The heatmap displays the –log10(p-values) of types of mapped non-coding RNA enrichments (rows) for 21 worm-fly conserved modules (columns) using the hypergeometric test.
